# Alternative mRNA Splicing: Control by Combination

**DOI:** 10.1371/journal.pbio.0030406

**Published:** 2005-10-25

In 1977, a flurry of papers ushered in a radical new concept in molecular biology—the idea of RNA splicing. It had been known for some years that the information for building organisms is stored as DNA sequences, which are transcribed into messenger RNAs (mRNAs) before translation into proteins. Although it had been established that the DNA and mRNA sequences line up exactly in bacteria, molecular biologists began to suspect in the mid-1970s that the genomes of eukaryotes (organisms with nuclei) are organized somewhat differently. Eukaryotic genes, it turns out, are encoded in small sections scattered over enormous distances of DNA. To make proteins from these “split genes,” the whole length of DNA is transcribed into pre-mRNA and then converted into mRNA by spliceosomes—molecular machines that remove the non-coding pieces of RNA (the introns) and splice together the protein-coding pieces (the exons).

One important consequence of RNA splicing is that one gene can produce several different mRNA variations, or isoforms, simply by stitching together different combinations of exons. For example, a single gene in vertebrates encodes calcitonin (a thyroid hormone that controls calcium levels) and calcitonin-gene-related peptide (a neuropeptide). Alternative splicing also contributes to human disease—for instance, the selection of different splice sites generates aberrant ratios of mRNA isoforms in several neurological diseases.

But how are these alternative splice sites selected? One popular model proposes that alternative splicing in mammalian cells is largely controlled by binding of general splicing factors to pre-mRNA molecules during the formation of the spliceosome. The spliceosome contains many of these factors, including a class of proteins called SR proteins, which contain one or two RNA-binding domains and a protein–protein interaction domain that is rich in serine and arginine amino acids. An important prediction of the combinatorial model for control of alternative splicing is that alternatively spliced transcripts will recruit different combinations of pre-mRNA splicing factors in vivo. New data from Mabon and Misteli support this prediction.

Pre-mRNA splicing factors accumulate at sites of active transcription, and splicing and can be detected and quantified in individual living cells by tagging the splicing factors with fluorescently labeled antibodies. So, to see whether different factors accumulate at alternatively spliced transcripts, the researchers developed stable cell lines carrying versions of the gene encoding a protein called tau designed to splice in different ways. In healthy people, exon 10 of the *tau* gene is included or excluded from *tau* mRNA with roughly equal probability during pre-mRNA splicing; in people with a rare Parkinsonism-like neurological disorder, mutations near one end of exon 10 result in its predominant inclusion. The researchers, therefore, examined cell lines carrying *tau* genes with and without a mutation of this type to change the ratios of mRNA transcripts including or excluding exon 10. Their results show that a subset of SR protein splicing factors is efficiently recruited to *tau* transcription sites that produce exon 10–containing mRNA, but less efficiently recruited to transcription sites where exon 10 is excluded.

These results provide the first in vivo evidence for the differential association of pre-mRNA splicing factors with alternatively spliced transcripts, and support a combinatorial mechanism for spliceosome formation. Exactly which splicing factors are recruited to each spliceosome would depend on both the concentration of each factor in individual cell types and the regulatory elements present in each pre-mRNA. The combinatorial mechanism for the control of alternative splicing, the authors suggest, could allow cells to adjust splicing outcome (and consequently which proteins they express) rapidly in response to intracellular or extracellular cues, as well as contributing to the generation of protein diversity. —*Jane Bradbury* [Fig pbio-0030406-g001]

**Figure pbio-0030406-g001:**
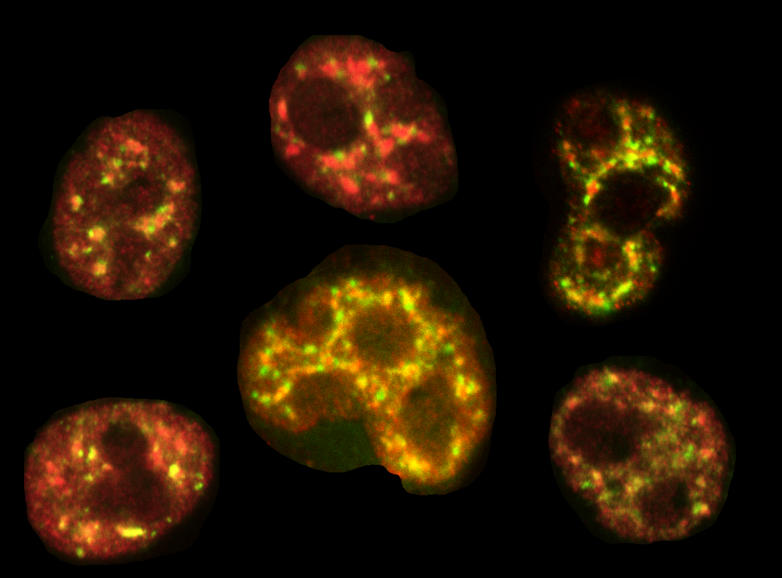
Pre-mRNA splicing factors (red) are recruited to sites of transcription and splicing (green) in the nucleus of mammalian cells

